# Induction of Regulatory Properties in the Intestinal Immune System by Dimethyl Fumarate in Lewis Rat Experimental Autoimmune Neuritis

**DOI:** 10.3389/fimmu.2019.02132

**Published:** 2019-09-10

**Authors:** Kalliopi Pitarokoili, Hussein Bachir, Melissa Sgodzai, Thomas Grüter, Steffen Haupeltshofer, Alexander Duscha, Xiomara Pedreiturria, Jeremias Motte, Ralf Gold

**Affiliations:** Department of Neurology, St. Josef Hospital, Ruhr-University Bochum, Bochum, Germany

**Keywords:** experimental autoimmune neuritis (EAN), chronic inflammatory demyelinating polyneuropathy, dimethyl fumarate (DMF), GALT (gut associated lymphoid tissue), autoimmunity

## Abstract

**Objective:** Dimethyl fumarate (DMF) exerts immunomodulatory and neuroprotective effects in the animal model of experimental autoimmune neuritis (EAN) in the Lewis rat. DMF has been shown to modulate gut microbiota in veterinary medicine, however the effects of oral DMF on the gut-associated lymphoid tissue (GALT) remain unknown.

**Methods:** Lewis rats were treated orally twice daily with DMF up to day 10 after immunization with immunogenic P2 peptide. Histological, flow cytometric and RT-PCR analyses of the GALT (intraepithelial layer, lamina propria, and Peyer patches) in duodenum, jejunum, and ileum were performed *ex vivo*. Moreover, cell transfer experiments were used to examine the protective effects of GALT regulatory T cells of the Peyer patches.

**Results:** In the upper layers of duodenum, DMF induced a reduction of the toll-like receptor 4 (TLR4) mRNA expression. This was combined by a decrease of the pro-inflammatory lamina propria IFN-γ mRNA expression. In the ileum, we detected an immunoregulatory phenotype characterized by an increase of FoxP3 mRNA expression and of the nuclear factor (erythroid-derived-2)- like 2 (Nrf2) downstream molecule heme oxygenase-1 (HO-1) mRNA. Finally, CD4^+^ CD25^+^ regulatory T cells were increased in the Peyer patches. *In vivo*, the protective effect of these regulatory cells was verified by cell transfer into recipient EAN rats.

**Conclusions:** Our results identified a novel immunomodulatory effect of DMF through the different regions and layers of the small intestine, which led to an increase of regulatory T cells, exerting a protective role in experimental neuritis.

## Introduction

Oral dimethyl fumarate (DMF) is approved since 2014 in Europe for the treatment of multiple sclerosis (MS) based on a wide spectrum of immunomodulatory and neuroprotective effects with a favorable safety profile (1, 2).

After oral intake and absorption in the small intestine, DMF, is rapidly hydrolyzed by esterases to its metabolite monomethyl fumarate (MMF) (3). Studies in experimental autoimmune encephalomyelitis (EAE), the mouse model for MS, revealed a neuroprotective mechanism of action through the activation of the transcription factor Nrf2 [nuclear factor (erythroid derived 2)-related factor 2] (4–8). Possible sites of action of Nrf2 mediated mechanisms are dendritic cells (DC) as MMF-induced Nrf2 signaling inhibits DC maturation and DC-mediated T cell proliferation (9–11).

Furthermore, DMF and, more so, its active metabolite, MMF, are known agonists of the hydroxycarboxylic acid receptor 2 (HCAR_2_), a G protein coupled membrane receptor. Studies in *Hca2*^−/−^ mice proved that HCAR_2_ interferes with neutrophil adhesion to endothelial cells and chemotaxis whereas Parodi et al. characterized a HCAR2-dependent nuclear factor “kappa-light-chain-enhancer” of activated B-cells (NF-kB) -mediated anti-inflammatory effect on microglia *in vitro* (12, 13).

The immunomodulatory effects of DMF in the animal model of acute and chronic autoimmune, demyelinating diseases of the peripheral nervous system [Guillain-Barré syndrome (GBS) and chronic inflammatory demyelinating polyneuropathy (CIDP)] the experimental autoimmune neuritis (EAN) have been previously investigated from our group after neuritis induction with myelin protein P2 emulsified in complete Freund's adjuvant (CFA) (14, 15).

Preventive treatment with oral DMF at a concentration of 45 mg/kg twice daily, significantly ameliorates clinical neuritis, presenting with a weakness of all four extremities, by reducing demyelination and inflammatory infiltration in the sciatic nerves. The neuroprotective effects of DMF correlated with an increase of Nrf2 positive axons (16). These results were confirmed by Han et al., who showed that in sciatic nerves, DMF treatment elevated the level of Nrf2 and its target gene hemoxygenase-1 (HO-1), which could facilitate macrophage polarization toward M2 type (17). Furthermore, a direct neuroregenerative potential of DMF for the peripheral nerves was shown in a crushed-nerve model as it led to an upregulation of HO-1 in Schwann cells and possibly motor neurons (18).

A mode of action of DMF, which has not been investigated up to now, neither in the EAE nor in EAN are its effects on the gut associated lymphoid tissue (GALT). GALT is a crucial component of the immune system, capable of modulating autoimmune diseases as described for the mouse model of MS after an enriched diet with propionic acid, a short-chain fatty acid (19). The autoimmune neurological diseases MS, GBS, and CIDP are caused by a combination of genetic factors and environmental exposures and have increased in incidence in the past decades, suggesting an increase of environmental risk factors (20). A main site of interaction of immune system components with ambiental factors is the gut and our group has already shown that nutrients such as capsaicin, the active component of chili pepper, modulate the intestinal immune system in the context of EAN. After preventive diet enriched with capsaicin we detected a reduction of clinical neuritis signs in combination with an increase of the expression of the immunoregulatory macrophage marker F4/80^+^ in the lamina propria (21).

DMF also seems to exert direct effects on intestinal microbiota. Ma et al. have shown that DMF detoxified mycotoxins in the intestine of BALB/c mice model and increased the diversity of gut microbiota (22).

Whether, however, immunomodulatory factors, such as DMF directly influence GALT remains unknown. At the current study, we investigated the effect of orally administered DMF in EAN in the GALT. Thereby we investigated with histological, flow cytometric, and RT-PCR analyses the immunological alterations in all parts of the small intestine (duodenum, jejunum, ileum) as well as in all layers composing these parts (intraepithelial layer, lamina propria, and Peyer patches) and in the mesenteric lymph nodes at day 10 after immunization (induction phase, beginning of the clinical symptoms). We completed the investigation by a cell transfer of the cellular components of the Peyer patches, which showed an immunoregulatory potential. Our data reveal a novel mechanism of action for this potent immunomodulator.

## Materials and Methods

### Antigens

The neuritogenic P2 peptide, corresponding to amino acids 53–78 of rat myelin P2 protein, was synthesized by Dr. Rudolf Volkmer from Charité Universitätsmedizin (Berlin, Germany).

### Study Design—Disease Induction and Clinical Score Assessment

A total of 64 female Lewis rats, 6–8 weeks old from Charles River Co. (Sulzfeld, Germany) (weight 160–180 g) were used in the present study. They were anesthetized by 1.5–2.0% isoflurane in oxygen) and immunized by subcutaneous injection into the root of the tail 250 μg P2_53−78_ peptide in PBS, emulsified in an equal volume of CFA containing 1 mg/ml *Mycobacterium tuberculosis* H37RA (Difco, Detroit, MI). Weighing and scoring for disease severity was performed daily by two independent investigators until day 10 post immunization (p.i.) (begin of clinical symptoms). All experiments were reviewed and approved by the North-Rhine-Westphalia authorities for animal experimentation (TVA 84-02.04.2014-A451).

### *In vivo* Treatment With Dimethyl Fumarate

Dimethyl fumarate (Biogen Idec, Cambridge, USA) was dissolved in 0.08% methylcellulose in tap water and a total volume of 300 μl was administered twice daily by oral gavage starting from the day of immunization to day 10 p.i; control groups received 300 μl of methylcellulose 0.08% orally twice daily. Rats were kept under standardized, pathogen free conditions at the local animal facility, Medical Faculty, Ruhr-University, Bochum, Germany and food and water were provided *ad libitum*.

The animals were randomly divided into the following groups: control group treated with methylcellulose 0.08% in tap water (*n* = 4–5, for each experiment) and a 45 mg/kg body weight DMF-treated group (*n* = 4–5 for each experiment). The experiments for the histological, flow cytometric, and RT-PCR analyses were repeated two to three times as described below.

### Histopathological Assessment and Immunohistochemistry

After transcardial perfusion with PBS (Gibco) on day 10 p.i. the small intestine was isolated and dissected into its three parts (duodenum, jejunum, ileum). Intestinal parts of each compartment were washed with PBS and then kept in paraformaldehyde 4% (PFA) for 24 h. Dehydration was performed in a 30% sucrose solution for 24 h and then their segments were embedded in Tissue-Tek OCT Compound and snap-frozen in liquid nitrogen. For histopathology assessment, rat tissue was sectioned (8 μm) on a cryostat (Leica Biosystems) and mounted on glass slides (Hartenstein, Würzburg, Germany).

For the immunohistochemical staining, cryostat sections were fixated in acetone at 20°C for 10 min and were exposed to the mouse monoclonal antibodies (mAb) anti-rat 15-6A1 (Hycultec, Pan T-Cells CD3 1:100), anti-rat ED1 (Hycultec, anti-CD68, macrophages, 1:100), and anti-rat toll-like receptor 4 (TLR4) (1:100, AA49-247). Secondary antibodies conjugated with Alexa 555 (1:1000) or Alexa 488 (1:1000) (Thermo Fisher, Schwerte, Germany) were used according to manufacturer's protocol and DAPI (4′, 6′ diamino-2-phenylindole·2HCl, Biozol, Eching, Germany) was used for fluorescent staining of DNA. Fluorescent signals were detected using an inverted fluorescence microscope (BX51; Olympus, Tokyo, Japan) equipped with an Olympus DP50 digital camera.

For histopathological assessment and immunohistochemistry, slides were blinded by a not-involved third person and labeled with a numeric-code, which was unblinded after analysis.

The omission of the primary antibodies served as negative control. Specificity of the staining was also controlled on sections of peripheral lymphoid organs.

### Isolation of Mononuclear Cells From the Lamina Propria, Peyer Patches, and Lymph Nodes and FACS Analyses

The isolation of mononuclear cells from lamina propria was performed as described by Weigmann et al. (23). Briefly the intestine was removed from mesenteric fat tissue and Peyer patches were excised. The small intestine was then opened longitudinally and cut into pieces. After incubation with EDTA and dithiothreitol (DTT) in Hanks' balanced salt solution (HBSS) (predigesting solution 1x HBSS containing 5 mM EDTA and 1 mM DTT), vortexing and passing through a cell strainer, the suspension of epithelial cells, villus cells, subepithelial cells, and intraepithelial layer (IEL) cells were removed. The remaining lamina propria (LP) with muscle layer was collected. After three incubations with digesting solution at 37°C for 20 min under slow rotation containing collagenases, DNases, and dispases [Digesting solution 0.05 g of collagenase D (Roche), 0.05 g of DNase I (Sigma), and 0.3 g of dispase II (Roche) in 100 ml of 1x PBS], the suspension was subjected to Percoll—gradient separation. After the cells were collected, they were stained with the antibodies described on the specific methods and analyzed using fluorescence-activated cell sorting (FACS).

Furthermore, the mesenteric lymph nodes (mLNs) were removed after transcardial perfusion with PBS (Gibco) on day 10 p.i. under aseptic conditions. Single mononuclear cell preparations from Peyer patches (PPs) and mesenteric lymph nodes were stained using conventional flow cytometry methods (*n* = 4–5/group for each experiment).

We evaluated the frequency of CD4^+^ T cells, CD11b^+^ cells, MHCII^+^ cells, CD11b^+^ MHCII^+^, CD4^+^CD25^+^ regulatory cells, and CD4^+^ CD25^+^ FoxP3^+^ regulatory T-cells expression by FACS staining (eBioscience, San Diego, CA). FACS analyses were performed with a FACS Canto II (BD Pharmingen, Heidelberg, Germany) and FlowJo software (Tree Star). Monoclonal antibodies were used to detect CD4-FITC (1:500, eBioscience), CD11b-PE (1:200, BD Bioscience), CD25-APC (1:100, eBioscience), and MHC-II-Alexa647 (1:300, Bio-Rad) in accordance with the manufacturer's instructions.

### Tissue Preparation, RNA Isolation, and Gene Expression Analyses With Quantitative RT-PCR

Total RNA was isolated from the intestinal wall (IEL+LP) of each part of the small intestine (duodenum, jejunum, ileum) as well as from the mesenteric lymph nodes of rats at day 10 p.i. (*n* = 4/group for each experiment) using the RNeasy Mini extraction kit (Qiagen, Hilden, Germany). All samples were treated with the RNA Stabilization Reagent (RNAlater, Qiagen, Hilden, Germany) and stored at −80°C until use. Total RNA was reverse-transcribed into cDNA according to the manufacturer's protocol for the Reverse Transcription System (Promega, Madison, WI).

Sequence-specific primers and probes were designed and sequences are presented below, sen (sense), ase (anti-sense), then mRNA expression levels were analyzed by quantitative RT-PCR according to the manufacturer's instructions (Quanta Biosciences, VWR, Germany). Sequence-specific primers and probes were designed using the genome browser database from (University of California; http://genome.ucsc.edu/) and a web program Primer3 (http://bioinfo.ut.ee/primer3-0.4.0/).

TLR4 (sen GCGCCTAAAACCCATTATGTT, ase TGATTCTTTGCCTGAGTTGCT), Nrf2 (sen CTC TCT GGA GAC GGC CAT, ase CTG GGC TGG GGA CAG TGG), HO-1 (sen CTG GGC TGG GGA CAG TGG, ase GAA AAG AGA GCC AGG CAA GAT), NQO1 (sen GTT TCT TTT TCC CCA GTT TGC, ase GGC TAC ACC TCT CCC TGA TTC), F4/80 (sen CAG CTG TCT TCC CGA CTT TC, ase TAA TCA AGA TTC CGG CCT TG), FoxP3 (sen AGG CAG AGG ACA CTC AAT GAA, ase ACT GCT CCC TTC TCA CTC TCC), IFN-γ (sen AAA GAC AAC CAG GCC ATC AG, ase CTT TTC CGC TTC CTT AGG CT), IL-10 (sen CCT GCT CTT ACT GGC TGG AG, ase TCT CCC AGG GAA TTC AAA TG), and IL-4 (sen TGA TGG GTC TCA GCC CCC ACC TTG C, ase CTT TCA GTG TTG TGA GCG TGG ACT C). RT-PCR amplifications were carried out using the real-time PCR System 7500 (Applied Biosystems) using the protocol recommended for the GoTaq qPCR Master Mix (Promega, Germany). In this context the relative expression ratio is calculated only from the RT-PCR efficiencies and the crossing point deviation of the sample vs. a control a method described by Pfaffl (24). β-Actin and GAPDH were used to normalize relative mRNA expression. Each experiment was performed in duplicate and the mean Ct was used in the equation.

### Cell Transfer Experiments, Electrophysiological, Histological, and RT-PCR Analyses

Cell transfer experiments were performed twice (*n* = 10 for each group for the donor groups, *n* = 5 for each group for the recipient groups).

Total cell preparations from Peyer patches were isolated from DMF- or vehicle-treated rats after 10 days of DMF 45 mg/kg or vehicle-treatment. After isolation, the cells were resuspended in sterile PBS and injected intravenously into recipient EAN rats at induction phase of EAN (day 7 p.i.). Three million cells were transferred intravenously to each recipient rat in 300 μl PBS.

Disease severity was assessed employing a scale ranging from zero to 10 originally described by Enders et al. (25): 0 normal; 1 less lively; 2 impaired righting/limb tail; 3 absent righting; 4 atactic gait, abnormal position; 5 mild paraparesis; 6 moderate paraparesis; 7 severe paraplegia; 8 tetraparesis; 9 moribund; 10 death.

Nerve conduction tests were performed by a blinded investigator on the day before immunization (−1) and on day 18 (maximum of natural disease course) p.i. The rats were anesthetized intraperitoneally (i.p.) with xylazine and ketamine (10 and 50 mg/kg, respectively). Using a fully digital recording Keypoint apparatus (Dantec, Skovlunde, Denmark) and paired needle electrodes inserted into the sciatic notch (hip; proximal) or the popliteal fossa (distal), the sciatic nerve was stimulated with supramaximal rectangular pulses of 0.05 ms duration and the resulting compound muscle action potential (CMAP) was recorded from needle electrodes placed subcutaneously over the dorsal foot muscles. A ground electrode was placed between the distal stimulating electrode and the active recording electrode. To calculate the motor nerve conduction velocity (MNCV), the distance between stimulating cathodes was divided by the difference of the latency (26, 27). Temperature differences were minimized by conducting the study as soon as the anesthesia had taken effect and by warming the leg with a heating lamp.

Recipient rats were sacrificed at day 26 p.i. The sciatic nerves were dissected and analyzed histologically for CD3^+^ T cells and CD68^+^ macrophages as described above. Furthermore, RT-PCR analyses for the anti-inflammatory (IL-4) and proinflammatory (IFN-γ) cytokines were performed in the peripheral nerves as described above.

### Statistical Methods

Statistical analyses were performed by GraphPad Prism 7 software (GraphPad Software Inc., San Diego, USA). Data are provided as mean ± SD (standard deviation). Differences between pairs of groups were tested by Student's *t*-test. In all experiments, a *P* value of < 0.05 was defined as statistically significant, and *P* < 0.0001 was considered as highly statistically significant.

## Results

### DMF Treatment Reduces TLR4 Expression and Induces an Anti-inflammatory Milieu in the Upper Layer of Duodenum

#### Duodenum

We initially investigated the effects of oral DMF on the upper layers of the duodenum (the IEL and LP), which first come in contact with DMF. As these areas are highly enriched with dendritic cells, that recognize antigens through the expression of pattern recognition receptors (such as Toll-like receptors, TLRs); we investigated the expression of TLR4 mRNA expression and histological distribution as an abundant TLR, activated mostly through lipopolysaccharide and leading to an NF-kB-mediated activation of the innate immune system.

**Figure 1:**
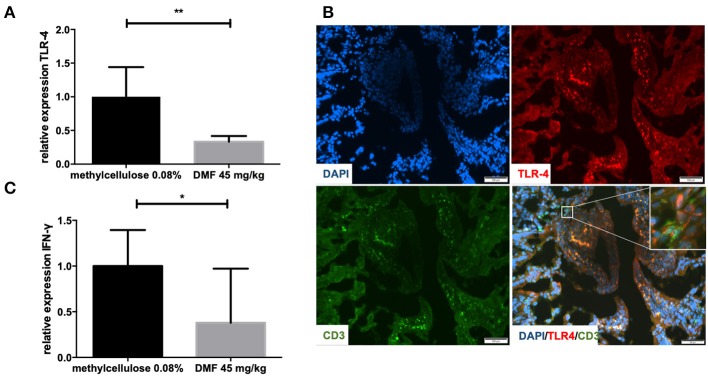
**(A)** Relative expression of Toll-like receptor-4 (TLR-4) expression in the upper layers of the small intestine (intraepithelial layer and lamina propria) in duodenum showing a decrease of its expression after 10 days of treatment with oral 45 mg/kg dimethyl fumarate. The experiment was repeated twice with similar results (*n* = 4/group, ^**^p < 0.001). **(B)** Representative pictures of immunohistochemical staining in the duodenum of Lewis rat at day 10 p.i. for TLR4, CD3 T cells, and DAPI (nuclear staining) showing the expression of TLR4 mostly in the intraepithelial layer and in close contact with CD positive cells (micrograph). Scale bars indicate 100 μm. **(C)** Relative expression of interferon-gamma (IFN-γ) expression in the upper layers of the small intestine (intraepithelial layer and lamina propria) in duodenum showing a decrease of its expression after 10 days of treatment with oral 45 mg/kg dimethyl fumarate. The experiment was repeated twice with similar results (*n* = 4/group, ^*^p < 0.05).

Peyer patches and muscular layers of the small intestine were removed and TLR4 mRNA expression was investigated in the remaining IEL and LP. TLR4 mRNA levels were reduced in the IEL and LP (representative data of one experiment, repeated twice with similar results, 45 mg/kg DMF- vs. vehicle-treated-groups *n* = 4/group ^**^p < 0.001, Figure 1A). In order to detect the localization of TLR4 expression in the IEL and lamina propria we performed immunohistochemical analyses. TLR4 was found at the upper layer of the LP and IEL in close contact with CD3 T cells (Figure 1B).

Regarding cytokine mRNA expression in these upper layers we found a decrease of IFN-γ expression, which implies the induction of an anti-inflammatory milieu (representative results of one experiment, performed twice with similar results, 45 mg/kg DMF- vs. vehicle-treated-groups *n* = 4/group ^*^p < 0.05, Figure 1C).

We found no differences in the mRNA expression of Nrf2 or its downstream molecules heme oxygenase-1 (HO-1) and NAD(P)H-quinone oxidoreductase 1 (NQO-1) as well as macrophage marker F4/80 in the upper layers of the duodenum (experiment repeated three times, data not shown).

#### Jejunum and Ileum

**Figure 2:**
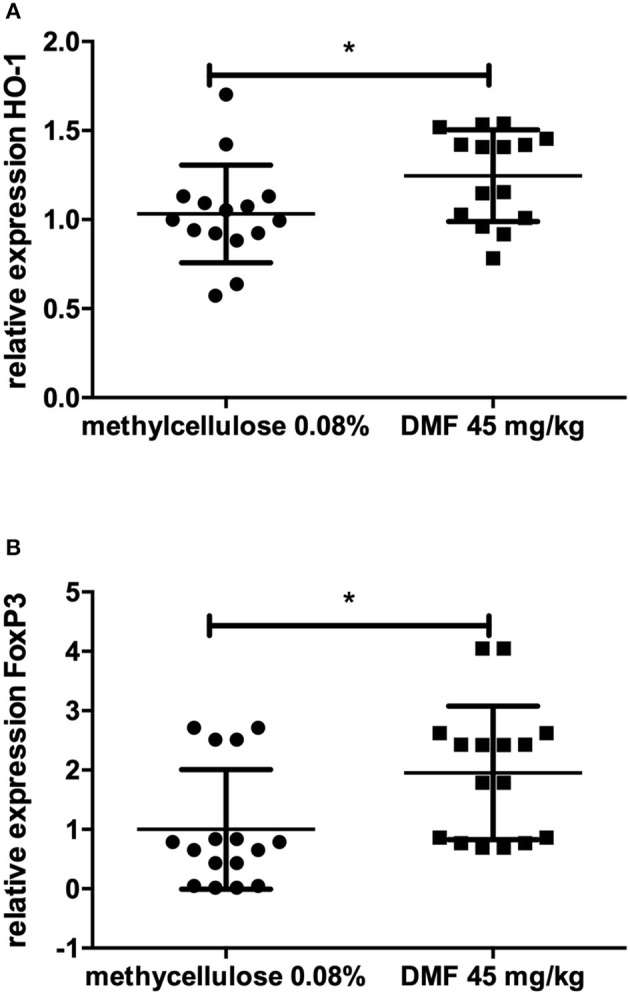
Relative expression of **(A)** heme oxygenase 1 (HO-1), the downstream molecule of nuclear factor E2-related factor 2 (Nrf2) and **(B)** regulatory molecule FoxP3 in the upper layers of ileum (intraepithelial layer and lamina propria) showing an increase of its expression after 10 days of treatment with oral 45 mg/kg dimethyl fumarate. The experiment was repeated twice with similar results (pooled results, *n* = 8/group, ^*^p < 0.05).

No further difference in the expression of TLR-4, IFN-γ, IL-10, IL-4, TNF-α, F4/80, Nrf2, or its downstream molecules was found in jejunum. In ileum, however, we found an increase of FoxP3 expression (experiment repeated twice, pooled data, mean values for 45 mg/kg DMF- vs. vehicle-treated-groups *n* = 8/group ^*^p < 0.05, Figure 2A) and HO-1 increase (experiment repeated twice, pooled mean values for 45 mg/kg DMF- vs. vehicle-treated-groups *n* = 8/group, ^*^p < 0.05, Figure 2B), Nrf2 showed again no differences in IEL and LP of ileum. F4/80 expression was not altered.

### Flow Cytometric Analyses in Lamina Propria Isolated Immune Cells

We proceeded to investigate the immune cell populations, which could mediate this anti-inflammatory action in the lamina propria in duodenum and the immunoregulatory action in ileum as well as their activation status.

We stained for CD4^+^, CD11b^+^, and MHCII^+^ positive cells. These cells showed no statistically significant differences in all parts of the small intestine.

### Flow Cytometric Histological and RT-PCR Analyses in the Peyer Patches

We then proceeded to investigate if the immunomodulatory actions of DMF in the IEL and LP could also be detected in the PP.

**Figure 3:**
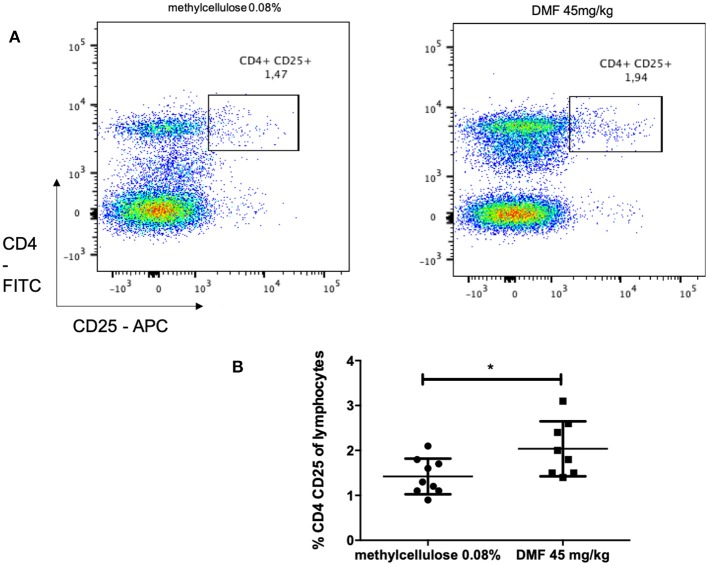
Flow cytometric analyses for CD4^+^ CD25^+^ T cells in the Peyer patches of the small intestine [**(A)** representative analyses for methylcellulose 0.08% treated on the left and DMF treated groups on the right] showed **(B)** a CD4^+^ CD25^+^ T cells increase after 10 days of treatment with oral 45 mg/kg dimethyl fumarate. The experiment was repeated twice with similar results (pooled results, *n* = 6/group, ^*^p < 0.05).

Indeed, we found an increase of CD4, CD25 regulatory cells in the PP (pooled data of two experiments with similar results, mean values of % CD4^+^CD25^+^ cells of CD4^+^ cells, vs. vehicle-treated-groups vs. 45 mg/kg DMF-treated group, *n* = 6/group 1.4 ± 0.3% vs. 2.0 ± 0.6%, ^*^p < 0.05, Figures 3A,B).

We also investigated the following populations: CD11b^+^ macrophages, MHCII^+^ lymphocytes, CD4^+^CD25^+^FoxP3^+^ regulatory T cells in three independent experiments but we did not find any significant differences.

We furthermore analyzed the mRNA expression of Nrf2 as well as FoxP3, TLR4, F4/80, IL-4, IL-10, IFN-γ, TNF-α in the Peyer patches but we did not find any significant differences to vehicle treated rats. Histological analyses confirmed an expression of Nrf2 on T cells in the lamina propria but not in the Peyer patches (Supplementary Figure 1).

### Immune Population in the Mesenteric Lymph Nodes

We proceeded to investigate further the immunological cascade initiated by DMF treatment in the draining lymph nodes of the intestine (mesenteric lymph nodes) at the induction phase of the disease (day 10 p.i.).

Using the mesenteric lymph node cells, we performed flow cytometric analyses looking for differences between following populations: CD4^+^ T cells, CD11b^+^ cells, MHCII^+^ cells, CD11b^+^MHCII^+^ dendritic cells, CD4^+^CD25^+^ cells. There were no statistically significant differences comparing DMF 45 mg/kg and vehicle-treated groups.

We also performed RT-PCR analyses for Nrf2 and its downstream molecules and for IL10, IFN-γ, and FoxP3 (performed twice). We found no statistically significant differences between DMF 45 mg/kg and vehicle-treated groups.

### Cell Transfer Experiments and Clinical Findings

As mentioned above, we found an increase of the possibly immunoregulatory CD4^+^CD25^+^ population in the PP. Therefore, we proceeded to investigate their functional role in a model of adoptive transfer. We isolated whole cell solutions from the PPs after 10 days of DMF treatment with 45 mg/kg or vehicle treatment and injected them intravenously in EAN rats at day 7 p.i. with P2_53−78_ (shortly before the begin of clinical symptoms). The experiment was repeated twice with similar results (*n* = 10/group for the donor groups, *n* = 5 for the recipient groups).

**Figure 4:**
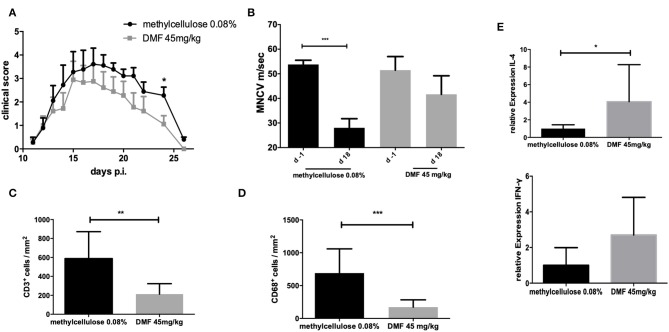
**(A)** Clinical course of EAN rats (*n* = 10/group) after immunization with P2 peptide at day 0 p.i. and transfer of Peyer patches (PP) cells from methylcellulose 0.08% or DMF-treated rats on day 7 p.i. Mean values and SEM are depicted for two experiment (pooled results). Recovery phase was characterized by improved EAN signs. On day 24 p.i. the mean clinical score for the group receiving PP cells from DMF treated rats was significantly reduced (^*^p < 0.05). **(B)** Motor nerve conduction velocity of the sciatic nerve one day before immunization (d-1) and at the peak of disease (day 18 p.i.) (*n* = 5/group): After proximal and distal stimulation of the sciatic nerve the conduction velocity was calculated. A statistically significant reduction of the MNCV appeared only for the group, which received the vehicle-treated cells but no difference in the MNCV was seen for the group receiving PP cells from DMF treated rats, indicating a protective role of these cells against distal demyelination. Mean values and SEM are depicted, *p*-values ^**^p < 0.0001. The experiments were repeated two times with *n* = 5/group. **(C)** Mean numbers of **(C)**. CD3^+^ and **(D)** CD68^+^ macrophages per mm^2^ sciatic nerve sections as measured by immunohistochemistry on day 18 p.i. from EAN rats (*n* = 5/group) after intravenous injection of PP cells from methylcellulose and DMF treated rats at day 7 post immunization. Mean values and SEM are depicted, *p*-values ^*^p < 0.05, ^**^p < 0.001, ^***^p < 0.0001. The experiment was repeated twice with similar results. **(E)** Relative expression of anti-inflammatory (IL-4) and proinflammatory (IFN-γ) cytokines in the sciatic nerves at the peak of disease (day 18 p.i.) showing a shift toward Th2 anti-inflammatory cytokines after intravenous injection of PP cells from methylcellulose and DMF treated rats at day 7 post immunization. The experiment was performed once (*n* = 5/group).

The incidence of EAN for the control group was 100% and the groups receiving DMF-treated PPs-cells showed an incidence of 90%. Treatment with PP cells pre-treated with DMF 45 mg/kg accelerated recovery from EAN signs (vehicle-treated vs. DMF-treated PP-cells ^*^p < 0.05 on day 24, data pooled from two experiments) (Figure 4A). There was no statistically significant difference between mean values of body weight of vehicle-treated and 45 mg/kg treated PP-cells groups (Table 1).

**Table 1:** Summarized data of the clinical EAN after transfer of cells from Peyer patches of rats treated from day 0 to day 10 after immunization with methylcellulose 0.08% or DMF 45 mg/kg orally twice daily.

	**Incidence *n*/total**	**Incidence (%)**	**Mean day of onset**	**Mean max clinical score**	**Mean score day 24**
Methylcellulose 0.08%	10/10	100	12.7 ± 2.4	3.6 ± 2.0	2.2 ± 1.0
DMF 45 mg/kg	9/10	90	15.1 ± 6.3	3.3 ± 0.8	1.0 ± 1.0 *p < 0.05

### Electrophysiological Findings in Recipient Rats

To elucidate the effect of intravenous DMF 45 mg/kg-treated, we performed electrophysiological measurements of the sciatic nerve at the maximum of the clinical course (day 18 p.i.) as described in Materials and Methods.

The conduction velocity of the sciatic nerves (MNCV) was significantly reduced in the rats, who received the vehicle-treated PP cells (mean MNCV on day 18 p.i. 28.0 m/s vs. day −1 p.i. 53.8 m/s, ^***^p < 0.0001, *n* = 5), whereas no difference of the mean MNCV on day −1 was seen for the rats receiving DMF-treated PP cells (Figure 4B), indicating the protective role of these cells against distal demyelination at this time point.

### Histological Finding in Recipient Rats

Next, we showed that clinical improvement at recovery phase correlates with the reduction of inflammatory infiltration of the peripheral nervous system (PNS). Intravenous administration of DMF-treated PPs-cells on day 7 p.i. significantly reduced infiltration of macrophages and lymphocytes in the sciatic nerves compared to vehicle-treated control group (T cells infiltrated/mm^2^ vehicle-treated PP cells group vs. DMF 45 mg/kg treated group ^**^p < 0.001, *n* = 5, macrophages infiltrates vehicle-treated PP cells group vs. DMF 45 mg/kg treated group ^***^p < 0.0001, experiment repeated twice, Figures 4C,D).

### Cytokine mRNA Expression in the Sciatic Nerves of the Recipient Rats

Furthermore, a Th2 cytokine shift in the sciatic nerves was observed for the 45 mg/kg treated DMF PPs-cells at day 26 p.i., as IL-4 mRNA expression showed a statistically significant increase (Figure 4E, ^*^p < 0.05, experiment performed once).

## Discussion

**Figure 5:**
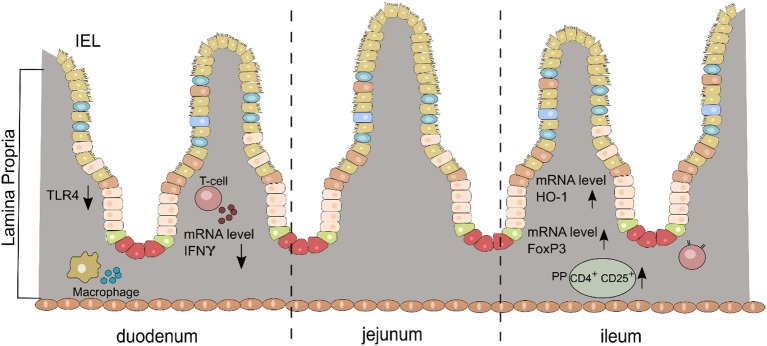
Schematic diagram of the anti-inflammatory/immunoregulatory effects of DMF in the small intestine (duodenum and ileum) for each layer (IEL, intraepithelial cells; LP, lamina propria; PP, Peyer patches).

We present for the first time an early and complex anti-inflammatory/immunoregulatory cascade in the small intestine after oral DMF treatment in the rat model of EAN (Figure 5).

We have chosen to investigate the changes in the GALT early in the induction phase of EAN as GALT is the first part of the immune system coming in close contact with DMF and its active metabolite MMF after oral application. We indeed found various changes in the GALT before any clinical neuritis signs, emphasizing the importance of the interaction of potent oral immunomodulatory drugs with GALT long before PNS effects are present.

Oral DMF treatment initially modulates Toll-like receptors on the upper layers of the duodenum, the first part of the small intestine. Experiments on pharmacokinetics indicate that DMF is almost completely hydrolyzed to MMF at an alkaline pH, but not at an acidic pH, suggesting that this hydrolysis occurs mainly within the small intestine and not in the stomach (3). MMF is then absorbed in the small intestine encountering dendritic cells and T cells of the upper layer of the GALT (28).

TLR4 is activated primarily by LPS and expressed on myelomonocytic subpopulations whereas other receptors such as TLR2 are expressed in peripheral blood monocytes (29). Histological staining showed TLR4 expression probably on DCs of the upper layers in close contact with CD3 cells, implying that a modulation of TLR4 expression (in our case a reduction) would influence directly activation status of CD3 cells. DCs are the sentinels of the immune system. The primary mechanism by which DCs recognize changes in the environment (e.g., foreign antigens) is the expression of pattern recognition receptors specific for evolutionarily conserved microbe-associated molecular patterns (29).

The mechanism mediating TLR4 expression reduction could be postulated as follows. Firstly, although not examined in this case, it has been previously reported that DMF modulates intestinal microbiota. Ma et al. have shown that DMF detoxified mycotoxin and improved the growth performance using the BALB/c mice model. 16S rRNA gene sequence analysis revealed that the richness and diversity of bacteria was increased after DMF treatment (22). Specifically, an increased abundance of bacteria producing short-chain fatty acids (SCFAs) was found in DMF group (22). SCFAs have previously shown to induce immunoregulatory cell populations in the small intestine (19). Therefore, the effect of DMF and microbiota modulation in EAN could be extremely relevant and has to be further investigated.

Furthermore, a direct effect on immune cells (for example macrophages of the GALT) could be mediated by the HCAR2 receptor, although further studies are needed to investigate the role of this receptor in the GALT (12, 13, 30).

Following TLR4 expression modulation we show a reduction of IFN-γ expression in these layers. LPS/TLR4 signaling can be separated into MyD88-dependent pathways, which mediate the activation of proinflammatory cytokine through NF-kB activation and MyD88-independent pathways, which mediate Type I interferon gene expression modulation (29). Type I interferons were not measured in our case, however the reduction of TLR4 expression could lead to an NF-kB activation and therefore reduction of the proinflammatory IFN-γ expression. A further differentiation of the individual populations of the lamina propria using flow cytometric analyses did not reveal any significant differences between the groups and as protein expression measurement of IFN-γ was not performed we can only postulate that the IFN-γ producing populations could be dendritic cells or lymphocytes of the lamina propria, whereas the biological significance of the increase of IFN-γ mRNA expression remains unknown.

Since we did not find any immunoregulatory alterations in duodenum, we proceeded to investigate jejunum and ileum. In ileum we indeed show an increase of FoxP3 mRNA expression together with an increase of HO-1 the downstream molecule of antioxidant/immunoregulatory Nrf2 pathway, previously involved in DMF signaling (6). Nrf2 gene expression was not increased in agreement with recent publications, which report a nuclear translocation of Nrf2 after DMF treatment rather than an increase of its expression (31, 32).

We then proceeded to investigate whether the previously described anti-inflammatory/immunoregulatory cascade could be followed toward the next part of the small intestine, the GALT tissue of the Peyer patches, which serve as “lymph nodes” of the enteric immune system with an “output” function toward the remaining immune system. The GALT is a unique immune compartment that is associated with the induction of regulatory cells, exerting a relevant immunoregulatory potential as it comprises 80% of the body's immune system. Previous publications mostly in the mouse models of MS show that the lymphoid tissues of the gut are specialized for the induction of regulatory cells, which can be directly responsible for the suppression of central nervous system—damaging encephalitogenic T cells (33).

In our case, we revealed in this rat model an increase of CD4^+^ CD25^+^ regulatory cells in the PPs. Intravenous cell transfer of whole PPs cell preparation from DMF and vehicle treated rats (treatment for 10 days) during the induction phase of EAN (day 7 p.i.) resulted in amelioration of the clinical EAN signs during the recovery phase. This correlated with a reduction of demyelination corroborated by electrophysiology and inflammatory infiltrates and an increase of IL-4 as an anti-inflammatory cytokine also at the recovery phase. A simultaneous although not significant increase of IFN- γ was also observed (Figure 4E). A role of IFN-γ on the production of different Th2 subpopulations has been indeed described before (34). Further clinical outcomes such as the disease incidence and duration did not differ significantly between the two groups. A possible shortcoming of our cell transfer experiment is that CD4^+^ CD25^+^ cells from the PPs were not sorted as whole PP lysates were injected; the effects on EAN parameters could have been more pronounced with sorted CD4^+^ CD25^+^ cells, which possibly show immunoregulatory functions. A further possibility is that the modulatory effects of these cells are more pronounced in the recovery phase in the context of neuroprotection as implied by the HO-1 increase in the ileum in this case and as reported before (18). Chronic EAN models are required in order to test this hypothesis. Furthermore, a further concept needing investigation is the influence of this regulatory cell population on the majority of the cells in Peyer patches, the B cells. However, although T cells are the minority of cells in the Peyer patches (15–20%), their immunomodulatory effect seems to be impressively important directly on EAN autoimmunity (35).

Returning to evidence from immunomodulatory treatment already used for autoimmune diseases, we have to point out that a regulatory role of multiple immunomodulatory drugs or antibiotics in the GALT has been already described. Ochoa-Repáraz et al. identified specific GALT-derived CD39 T regulatory cells in the mouse EAE model after teriflunomide treatment, another drug used for MS patients (35). Such CD39-T immunoregulatory cell populations in the GALT have also been described in mouse EAE after the repopulation that followed a treatment with anti-CD52 antibody, which is also a very potent immunosuppressive treatment for MS (36). There are no publications of intestinal immunomodulatory effects for DMF until now although mostly gastrointestinal side-effects have been described in a variety of clinical studies (37, 38).

Our data introduce for the first time a novel concept of action for DMF even though further investigation is needed to enlighten this pathway in further detail. Questions that remain open are the effect of DMF on microbiota and the role of HCAR2 receptor. Furthermore, the reason for the differential effects on GALT in duodenum, jejunum and ileum could imply an “immunological gradient” in the small intestine, which gradually modulates GALT but a similar effect on autoimmune diseases to our knowledge has not been defined before. Duodenum, jejunum, and ileum are however characterized by different microbiota colonization and therefore different T cell differentiation environment, which could explain the effects seen in our model (22, 39, 40). This study points out the need to evaluate GALT in each part of the small intestine as a unique immunological unit in order to understand the effects of oral immunomodulatory substances.

Our present results point out two important considerations for immunomodulatory drugs such as DMF:

- First, GALT modulation is a crucial aspect of their effect on autoimmune diseases as opposed to parenteral application of immunomodulation and should be investigated in the context of the different parts of the small intestine, each one as a unique immune microenvironment.
- Secondly, DMF initiates a surprising cascade of anti-inflammatory/immunomodulatory effects already during the first days of oral application, which have a direct impact on the control of EAN autoimmunity.

As CIDP treatment lacks novel immunomodulatory applications, DMF is a promising option not only because its effects after entering blood circulation but also because of GALT modulation.

## Ethics Statement

All experiments were reviewed and approved by the North Rhine-Westphalia, Germany authorities for animal experimentation (TVA 84-02.04.2017-A451).

## Author Contributions

KP: study design, acquisition of data, analysis and interpretation of data, and drafting and revising the manuscript for content. HB, MS, TG, XP, and JM: acquisition of data, analysis and interpretation of data, and revising the manuscript for content. SH and AD: acquisition of data, analysis and interpretation of data, and drafting and revising the manuscript for content. RG: study design and drafting and revising the manuscript for content.

### Conflict of Interest Statement

KP received travel funding and speaker honoraria from Biogen Idec, Novartis and Bayer Schering Pharma and funding from the Ruhr-University, Bochum (FoRUM-Program). TG received a travel reimbursement from Sanofi Genzyme and Biogen Idec, none related to this manuscript. JM received travel grants from Biogen idec, Novartis AG, Teva and Eisai GmbH, his research was funded by Klaus Tschira Foundation and Ruhr-University, Bochum (FoRUM-program), none related to this work. RG has received consultation fees and speaker honoraria from Bayer Schering, Biogen idec, Merck Serono, Novartis, Sanofi-Aventis, and TEVA. He also acknowledges grant support from Bayer Schering, Biogen idec, Merck Serono, Sanofi-Aventis and TEVA, none related to this manuscript. The remaining authors declare that the research was conducted in the absence of any commercial or financial relationships that could be construed as a potential conflict of interest.

## Supplementary Material

Supplementary Figure 1 Representative pictures of immunohistochemical staining in the duodenum of Lewis rat at day 10 p.i. for Nrf2 and CD3 T cells showing the expression of Nrf2 mostly in the lamina propria and not in the Peyer patches, this result was also confirmed by RT-PCR analyses, which did not find any Nrf2 induction or increase of its downstream molecules (HO-1, NQO-1) in the Peyer patches (arrows). Scale bars indicate 100 μm.

## Abbreviations

AUC: Area under curve
CFA: complete Freund's adjuvant
CIDP: chronic inflammatory demyelinating polyneuropathy
CMAP: compound muscle potentials
DAPI: 4′,6-Diamidin-2-phenylindol
DMF: dimethyl fumarate
DC: dendritic cell
EAN: experimental autoimmune neuritis
GALT: gut-associated lymphoid tissue
GBS: Guillain-Barré syndrome
HO-1: oxygenase-1
HCAR2: hydroxycarboxylic acid receptor 2
ICAM1: intercellular adhesion molecule 1
IEL: intraepithelial layer
IL: interleukin
IFN-γ: interferon-gamme
i.p.: intraperitoneally
LP: lamina propria
MNCV: motor nerve conduction velocity
mRNA: messenger ribonucleic acid
MHCII: major histocompatibility complex II
MMF: monomethyl fumarate
MS: multiple sclerosis
Nrf2: nuclear factor (erythroid derived 2)-related factor 2
NQO-1: NAD(P)H-quinone oxidoreductase 1
PBS: phosphate buffer saline
p.i.: post immunization
PNS: peripheral nervous system
PP: Peyer patches
TLR-4: Toll-like receptor-4
NF-kB: nuclear factor “kappa-light-chain-enhancer” of activated B-cells
RT-PCR: reverse transcriptase polymerase chain reaction
SCFA: small chain fatty acids.
