# Supplementary figures and images for: Induction of Regulatory Properties in the Intestinal Immune System by Dimethyl Fumarate in Lewis Rat Experimental Autoimmune Neuritis
Source: Front Immunol. 2019 Sep 10;10:2132. doi: 10.3389/fimmu.2019.02132 (PMC6746892; doi:10.3389/fimmu.2019.02132)

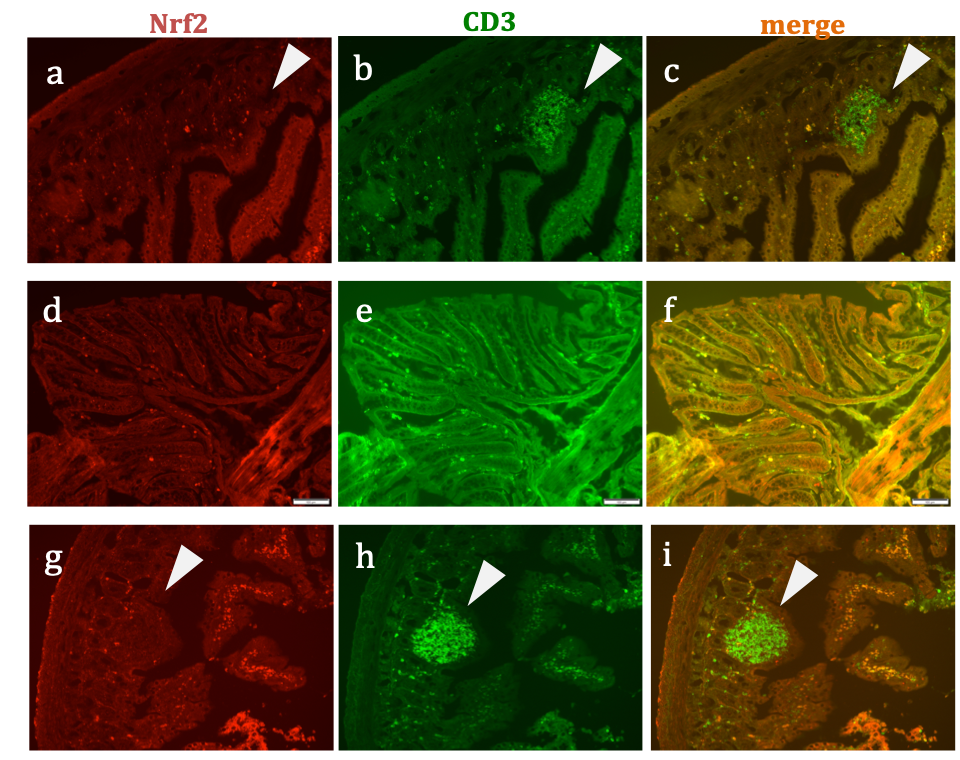

Supplement: Supplementary Figure 1 — Representative pictures of immunohistochemical staining in the duodenum of Lewis rat at day 10 p.i. for Nrf2 and CD3 T cells showing the expression of Nrf2 mostly in the lamina propria and not in the Peyer patches, this result was also confirmed by RT-PCR analyses, which did not find any Nrf2 induction or increase of its downstream molecules (HO-1, NQO-1) in the Peyer patches (arrows). Scale bars indicate 100 μm. [file Image_1.TIFF]
